# Intracellular replication of *Pseudomonas aeruginosa* in epithelial cells requires suppression of the caspase-4 inflammasome

**DOI:** 10.1128/msphere.00351-23

**Published:** 2023-08-17

**Authors:** Abby R. Kroken, Keith A. Klein, Patrick S. Mitchell, Vincent Nieto, Eric J. Jedel, David J. Evans, Suzanne M. J. Fleiszig

**Affiliations:** 1 Department of Microbiology and Immunology, Loyola University Chicago, Maywood, Illinois, USA; 2 Herbert Wertheim School of Optometry & Vision Science, University of California, Berkeley, California, USA; 3 Department of Microbiology, University of Washington, Seattle, Washington, USA; 4 College of Pharmacy, Touro University California, Vallejo, California, USA; 5 Graduate Groups in Vision Sciences, Microbiology, and Infectious Diseases & Immunity, University of California, Berkeley, California, USA; University of Kentucky College of Medicine, Lexington, Kentucky, USA

**Keywords:** *Pseudomonas aeruginosa*, inflammasome, cornea, keratitis, type three secretion system, epithelium, caspase-4, pyroptosis

## Abstract

Pathogenesis of *Pseudomonas aeruginosa* infections can include bacterial survival inside epithelial cells. Previously, we showed that this involves multiple roles played by the type three secretion system (T3SS), and specifically the effector ExoS. This includes ExoS-dependent inhibition of a lytic host cell response that subsequently enables intracellular replication. Here, we studied the underlying cell death response to intracellular *P. aeruginosa*, comparing wild-type to T3SS mutants varying in capacity to induce cell death and that localize to different intracellular compartments. Results showed that corneal epithelial cell death induced by intracellular *P. aeruginosa* lacking the T3SS, which remains in vacuoles, correlated with the activation of nuclear factor-κB as measured by p65 relocalization and tumor necrosis factor alpha transcription and secretion. Deletion of caspase-4 through CRISPR-Cas9 mutagenesis delayed cell death caused by these intracellular T3SS mutants. Caspase-4 deletion also countered more rapid cell death caused by T3SS effector-null mutants still expressing the T3SS apparatus that traffic to the host cell cytoplasm, and in doing so rescued intracellular replication normally dependent on ExoS. While HeLa cells lacked a lytic death response to T3SS mutants, it was found to be enabled by interferon gamma treatment. Together, these results show that epithelial cells can activate the noncanonical inflammasome pathway to limit proliferation of intracellular *P. aeruginosa*, not fully dependent on bacterially driven vacuole escape. Since ExoS inhibits the lytic response, the data implicate targeting of caspase-4, an intracellular pattern recognition receptor, as another contributor to the role of ExoS in the intracellular lifestyle of *P. aeruginosa*.

**IMPORTANCE:**

*Pseudomonas aeruginosa* can exhibit an intracellular lifestyle within epithelial cells *in vivo* and *in vitro*. The type three secretion system (T3SS) effector ExoS contributes via multiple mechanisms, including extending the life of invaded host cells. Here, we aimed to understand the underlying cell death inhibited by ExoS when *P. aeruginosa* is intracellular. Results showed that intracellular *P. aeruginosa* lacking T3SS effectors could elicit rapid cell lysis via the noncanonical inflammasome pathway. Caspase-4 contributed to cell lysis even when the intracellular bacteria lacked the entire T33S and were consequently unable to escape vacuoles, representing a naturally occurring subpopulation during wild-type infection. Together, the data show the caspase-4 inflammasome as an epithelial cell defense against intracellular *P. aeruginosa*, and implicate its targeting as another mechanism by which ExoS preserves the host cell replicative niche.

## INTRODUCTION

*Pseudomonas aeruginosa* is an opportunistic pathogen that can cause life- and vision-threatening infections. While often referred to as an extracellular pathogen, *P. aeruginosa* can adopt an intracellular lifestyle in various epithelial cell types, including corneal (1), bronchial (2), HeLa cells (3), and *in vivo* (4). The ability of *P. aeruginosa* to replicate inside a host cell depends on the type three secretion system (T3SS), with multiple roles played by the T3SS effector ExoS (5 – 7) encoded by only invasive strains (8). ExoS is a bifunctional protein with both RhoGAP and ADP ribosyltransferase (ADPr) activities. Broad substrate specificity of its ADPr domain (9, 10) enables ExoS to impact multiple host processes (11, 12). Demonstrated effects of ExoS include inactivation of host cell proliferative/survival signaling, disassembling the host cell cytoskeleton, freezing of intracellular membrane trafficking, and disruption of reactive oxygen species generation (9, 13 – 16). While the molecular components of host cells targeted by ExoS to enable *P. aeruginosa* to replicate intracellularly have not yet been elucidated, its ADPr activity is required, and the mechanisms shown include inhibition of autophagy (17) and evasion of lysosomes (18). Recently, we reported another role for the ADPr activity of ExoS in intracellular persistence: preservation of host cell viability (19). However, the mechanism of host cell death in the absence of ExoS ADPr activity is unknown, which was further explored here.

Eukaryotic host cells can sense and respond to intracellular pathogens (20, 21). Responses can include initiation of regulated cell death pathways (22), which may subsequently be suppressed by the bacteria to preserve their intracellular niche (23). One type of host response is pyroptosis, a form of inflammatory lytic cell death (20). Multiple pyroptotic pathways have been identified, each sensing different pathogen-associated molecular patterns (PAMPs) (24 – 27) or pathogen-associated aberrant activities (28, 29). The sensor converges on the activation of a cysteine-aspartic protease: caspase-1 for canonical inflammasome pathways (30, 31) and caspase-4/5 for later-discovered pathways designated noncanonical (32 – 34). Inflammatory caspases cleave and activate gasdermin D (35, 36), which assembles into pores in host plasma membranes (37). This leads to the release of cytokines interleukin-18 (IL-18) and IL-1β (38), and total lysis for many cell types (39).

*P. aeruginosa* effectors ExoS and ExoT can reduce IL-1β secretion in macrophages (40 – 42), suggesting an ability to interfere with inflammasome activation. Responses of macrophages and epithelial cells can differ. While inflammasomes are well studied in macrophages and other myeloid cells, the repertoire of inflammasomes in epithelial cells is usually limited, and their functions are less well studied (43). One inflammasome pathway studied in some corneal diseases is NLRP3 (44, 45), which can detect numerous stimuli (46) including ionic flux from pore formation (47), lysosomal damaging agents (48), and bacterial RNA (49). However, not all relevant studies verified which corneal cell type expressed NLRP3, or explicitly determined whether NLRP3 was activated, versus other inflammasome pathways that could also yield mature IL-1β. Recently, both caspase-4, which detects cytoplasmic LPS (50), and NLRP1, which detects pathogenic enzymatic activities and dsRNA (28, 51), were shown to be expressed and functional in the human corneal epithelium (52, 53). The Protein Atlas RNAseq data set for the corneal epithelial cell line hTCEpi (54) provides evidence for expression of caspase-4, caspase-5 [an additional LPS sensor (33, 55)], NLRP1, and NLRC4 (although its required sensor protein NAIP (56) was not detected) (57). While NLRP3, pyrin, and AIM2 inflammasomes were not detected in these cells, this does not preclude upregulation upon specific stimuli or *in vivo* (57).

Having shown that the T3SS effector ExoS inhibits rapid cell lysis induced by intracellular *P. aeruginosa* in corneal epithelial cells (19), we investigated the mechanisms underlying host cell death elicited and modulated by intracellular *P. aeruginosa*. In doing so, we also leveraged the finding that *P. aeruginosa* mutants lacking the entire T3SS (∆*exsA* mutants) remain toxic to corneal epithelial cells (58), contrasting with HeLa and CHO cells (59, 60). Results showed that caspase-4 is required for rapid corneal epithelial cell death in response to *P. aeruginosa* invasion, and in this way limits the accumulation of an intracellular population of bacteria. While caspase-4-dependent death occurred even if bacteria lacked the ability to leave vacuoles/enter the cytoplasm, the response was faster when they could. Moreover, the death response was enabled in otherwise unresponsive HeLa cells after stimulation with interferon gamma (IFN-γ), which has been shown to limit cytoplasmic subpopulations of *Salmonella* in a manner dependent on caspase-4 and GBP proteins (61). Since ExoS inhibits the cell death response to intracellular *P. aeruginosa*, these results implicate ExoS targeting a caspase-4-dependent response as another contributor to its well-established role in intracellular survival by *P. aeruginosa*.

## RESULTS

### *P. aeruginosa* lacking the T3SS kill corneal epithelial cells but not HeLa cells

**Fig 1:**
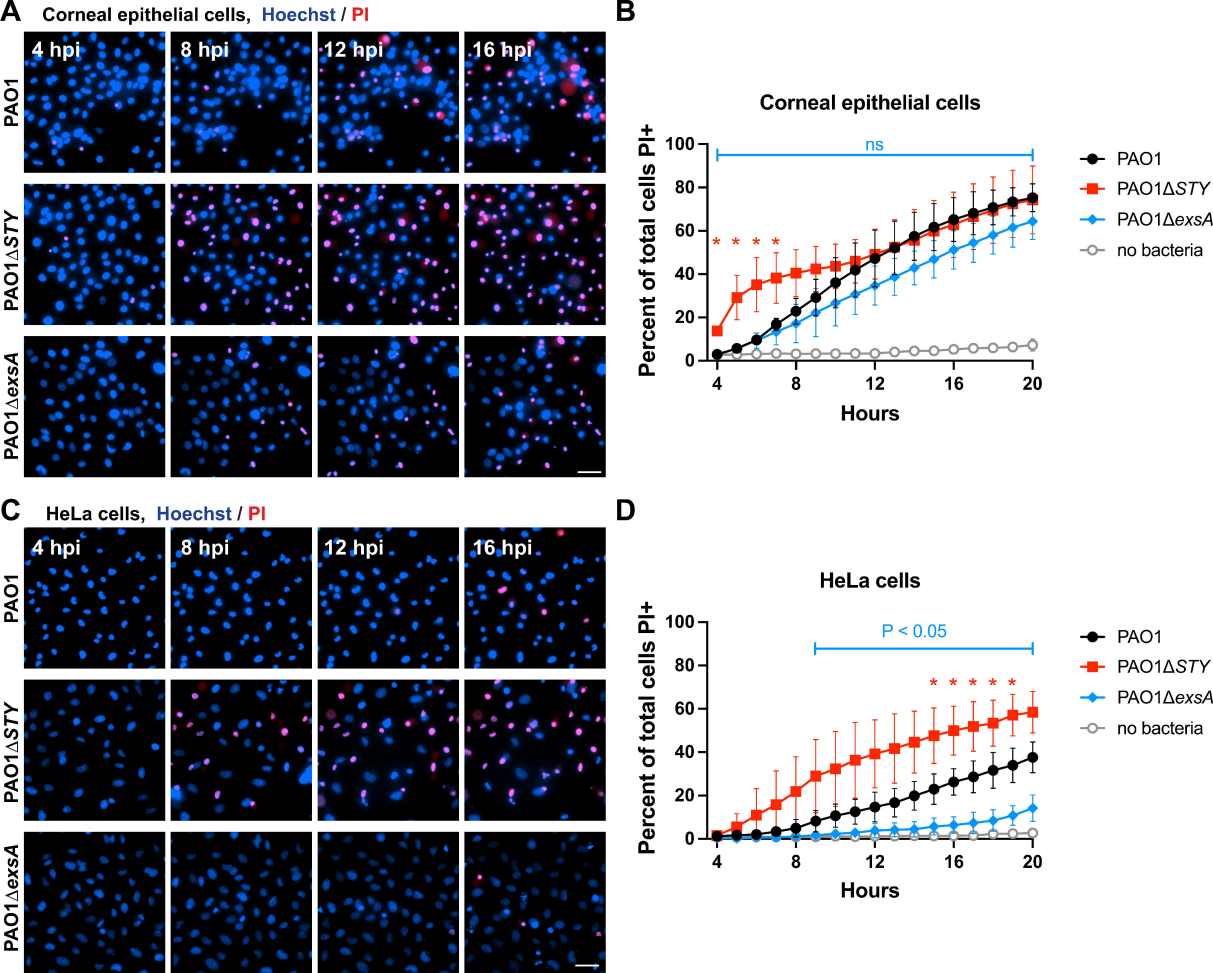
Host cell death rates from *P. aeruginosa* mutant infections. (**A**) Corneal epithelial cells (hTCEpi) were infected with indicated strain: wild-type PAO1, PAO1∆*exoSTY*, or PAO1∆*exsA* for 3 h at a multiplicity of infection (MOI) equal to 10. Hoechst (blue) was added to label nuclei. Non-associated bacteria were removed, and media with amikacin and propidium iodide (red) was added to kill extracellular bacteria. Cells were imaged hourly from 4 to 20 h post-infection. Select fields from indicated times are shown. Scale bar = 50 µm. (**B**) The percent of cells positive for propidium iodide at each timepoint was determined using a custom FIJI macro to segment all nuclei from the Hoechst channel, and all nuclei of dead cells in the propidium iodide channel. Multiple column *t*-tests were performed comparing wild-type PAO1 and PAO1∆*exsA*, and SD error bars are displayed from three replicates. (**C**) Experiment was performed identically as in panel A, but using HeLa cells. Scale bar = 50 µm. (**D**) Analysis was performed as in panel B, using the data obtained in HeLa cells.

Generally, it is thought that *P. aeruginosa* mutants missing the T3SS (i.e., ∆*exsA* mutants) do not kill epithelial cells, as shown for HeLa and CHO cells (59, 60). However, our published work has shown that ∆*exsA* mutants are able to kill corneal epithelial cells, with contributions made by the intracellular population (58). Recognizing that differences between cell types could assist in deciphering mechanisms, we compared corneal epithelial cells (hTCEpi) (54) to HeLa cells using the same methods. Impact of wild-type PAO1, ∆*exsA* mutants (lacking the entire T3SS), and ∆*exoSTY* mutants (lacking all known T3SS effectors) was studied. Prior studies have shown complementation of effectors ExoS and ExoT in these strains (19, 62), and ExsA complementation has also been demonstrated using chromosomal integration at a non-native site (19). After a 3-h invasion period, extracellular bacteria were eliminated using the non-permeable antibiotic amikacin (3). Cell death rates were measured with a FIJI macro that counts propidium-iodide-positive nuclei (permeabilized cells) and Hoechst-labeled nuclei (total cells) and reports a ratio over a 20-h post-infection period (19). The results confirmed that the two cell types were differentially susceptible. Death rates for corneal epithelial cells were statistically similar between wild-type and ∆*exsA* mutants (Fig. 1A and B), whereas HeLa cells infected with ∆*exsA* mutants showed very little cell death even at 20 h post-infection (Fig. 1C and D). In both cell types, the ∆*exoSTY* mutant yielded the most rapid cell death as noted previously (19). Thus, corneal epithelial cells have an intrinsic response to T3SS-null bacteria that is absent in HeLa cells, which may underly cell death occurring in response to T3SS-positive bacteria.

### Dynamics of cell death in bacterially occupied cells indicates roles for both T3SS machinery and T3SS effectors

Visual inspection of infected cells suggested that not all cells were occupied by bacteria, and that some nonoccupied cells also died. Recognizing that this could skew results in bulk analyses of cell death rates, we performed additional experiments to analyze only bacterially occupied cells. To enable this, we employed methods that allowed simultaneous detection of viable intracellular bacteria and the host cell (19). Wild-type (PAO1) was compared with mutants lacking the entire T3SS (∆*exsA* mutants), and ∆*exoSTY* mutants lacking only T3SS effectors, using quantitative time-lapse imaging over 20 h. Since ∆*exsA* mutants do not kill HeLa cells, this analysis was only done using corneal epithelial cells.

A method of detecting intracellular bacteria was needed, as amikacin-killed bacteria adhere to the dishes and cells, making intracellular bacteria impossible to distinguish if using constitutive fluorophore expression. To detect ∆*exsA* mutants inside cells, we previously used an arabinose-inducible green fluorescent protein (GFP) expression vector pBAD-GFP (58). This method involves induction of GFP expression using arabinose induction only after bacteria have invaded cells and extracellular populations are killed (using amikacin). To keep experimental methods consistent across samples, we explored the feasibility of using the induction method to also study wild-type and the ∆*exoSTY* mutants. In prior studies, these strains were visualized using a reporter for the T3SS (3), because T3SS effectors are needed for intracellular replication, and cytoplasmic populations of bacteria were uniformly T3SS positive (5, 19). Unfortunately, fewer bacterial cells exhibited cytoplasmic spread using the arabinose induction method compared to the T3SS reporter method (Fig. S1A). Moreover, while the host cell death rate was similar for wild-type PAO1 using both methods, it trended lower for ∆*exoSTY* mutants expressing arabinose-induced GFP (Fig. S1B). This suggested that GFP induction impacts the T3SS or some other factor relevant to cytoplasmic entry or spread: a critical step for intracellular infection by wild-type and ∆*exoSTY* mutants, but not for ∆*exsA* mutants (which remain in vacuoles [58]). Indeed, T3SS effector secretion was reduced *in vitro* using EGTA stimulation in the presence of arabinose when bacteria were also transformed with pBAD-GFP (Fig. S1C). Thus, the T3SS expression plasmid pJNE05 was used to study intracellular wild-type and ∆*exoSTY* mutants, reserving arabinose induction of GFP for the ∆*exsA* mutant. A limitation of this approach is that a subpopulation of intracellular wild-type bacteria can remain T3SS negative (58) and would be undetected in this analysis. In addition, high-level expression of GFP may impact other metabolic processes or virulence factors, although ∆*exsA* mutants with pBAD-GFP were shown to kill cells in a prior study (58). However, the advantage of this approach is that T3SS-dependent outcomes for intracellular bacteria can be spatially isolated and examined independently, and then compared to homogeneous populations of ∆*exsA* mutants missing the T3SS apparatus.

**Fig 2:**
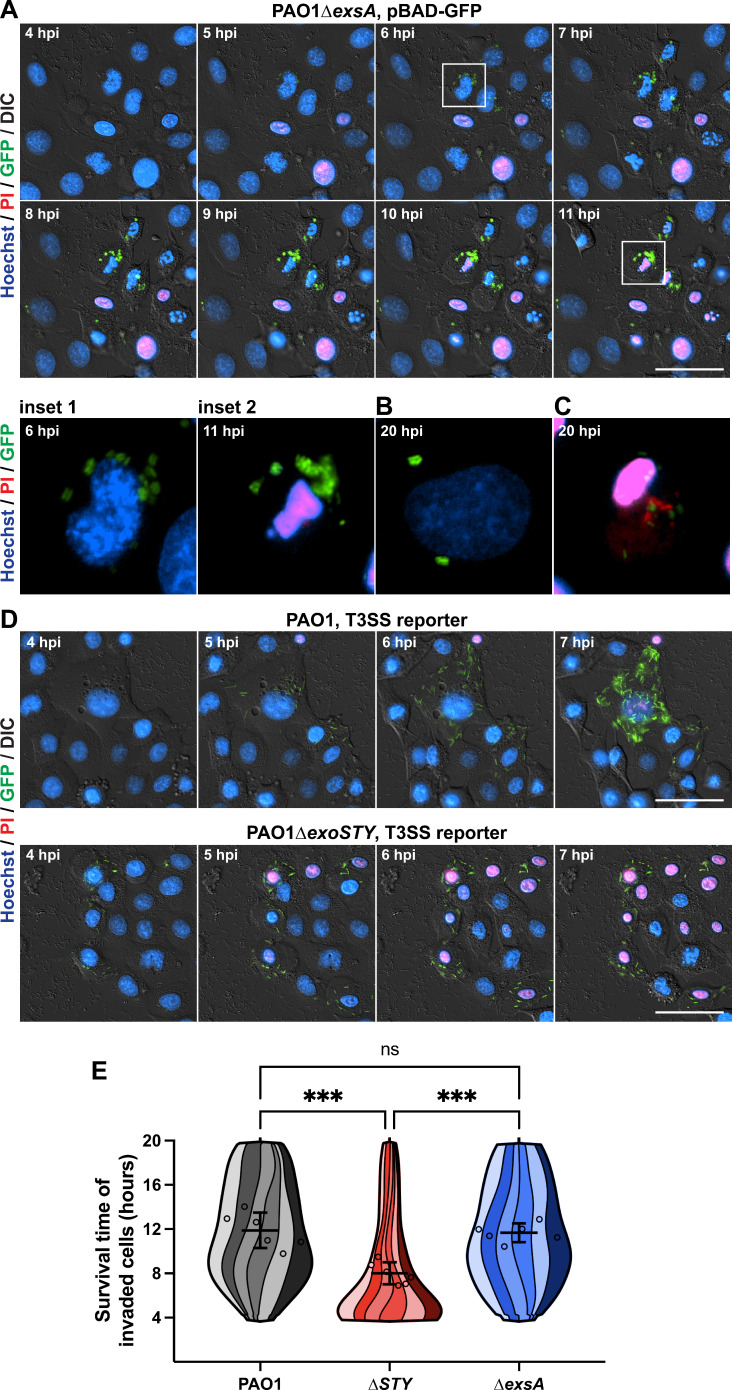
Intracellular ∆*exsA* mutants kill invaded cells at a similar rate to wild-type PAO1-invaded cells. (**A**) Bacteria were transformed with an arabinose-inducible GFP plasmid. Corneal epithelial cells (hTCEpi) were infected at an MOI of 10 and extracellular bacteria eliminated at 3 h post-infection using amikacin. A final concentration of 1% arabinose was used to induce GFP in surviving intracellular bacteria beginning at 3.5 h post-infection. Time-lapse imaging was conducted hourly from 4 to 20 h, and host cell nuclei detected with Hoechst and propidium iodide to determine time of death. Scale bar = 50 µm. Time-lapses are available as Movie S1. Insets 1–2 show the boxed cell at 6 and 11 h post-infection, respectively. (**B**) An example of a live invaded cell from 20 h post-infection. (**C**) Example of a dead invaded cell from 20 h post-infection, where the propidium iodide channel has been saturated such that propidium-iodide-positive bacteria can be visualized. (**D**) The T3SS-GFP reporter pJNE05 was used to visualize wild-type PAO1 and PAO1∆*exoSTY* infections, as described previously (19), to compare to PAO1∆*exsA* infections. Scale bar = 50 µm. Time-lapses are available as Movie S1. (**E**) A computational analysis approach was used to segregate only invaded cells and determine their survival time in hours. PAO1 condition includes 881 cells over six replicates; PAO1∆*exoSTY*, 967 cells; PAO1∆*exsA*, 862 cells. The survival times of all invaded cells were combined to a single super violin plot. Mean survival times with SD error bars are displayed, and significance is determined by one-way ANOVA. ***P < 0.005.

Representative images from a time-lapse experiment using ∆*exsA* mutants are shown in Fig. 2A. As expected, ∆*exsA* mutants localized to vacuoles inside the cells (58). Most bacteria-occupied cells died before the end of the 20-h assay, as shown by propidium iodide labeling of the nucleus. However, Fig. 2B shows an invaded cell still alive at 20 h (no propidium iodide labeling), representing a minority of cells at this timepoint. Videos of these time-lapse experiments are available in Movie S1, which includes cells visualized in Fig. 2B and C at 20 h post-infection. As shown previously, both wild-type PAO1 and ∆*exoSTY* mutant-infected cells (shown in Fig. 2D) replicated in the host cell cytoplasm after bacteria escape from vacuoles (19). In both cases, most invaded cells died by the end of the assay, the ∆*exoSTY* mutant doing so even more rapidly, expected due to lack of ExoS which normally counters cell death (19). Hours following host cell death, propidium iodide of intracellular bacterial bodies eventually replaced GFP signal (Fig. 2C; Movie S1).

A computational approach was used to measure when populations of invaded cells died (19), and a super violin plot visualization script developed by Kenny and Schoen was used to show independent replicates as stripes within each violin plot (63). This analysis examined 2,710 total invaded cells over six replicate experiments. Results confirmed that intracellular ∆*exsA* mutants killed their occupied cells at a broad range of timepoints, and the mean value of invaded cell survival times was unexpectedly similar to wild-type PAO1 (Fig. 2E). The analysis also confirmed that the intracellular ∆*exoSTY* mutant infections caused cell lysis at a more rapid rate, significantly different from both wild-type PAO1 and ∆*exsA* mutants (19). Thus, while cell death can be driven by vacuolar-contained bacteria, it was more rapid if bacteria could access the cytoplasm while lacking effectors.

### T3SS-null mutant bacteria engage nuclear factor-κB signaling in corneal epithelial cells

**Fig 3:**
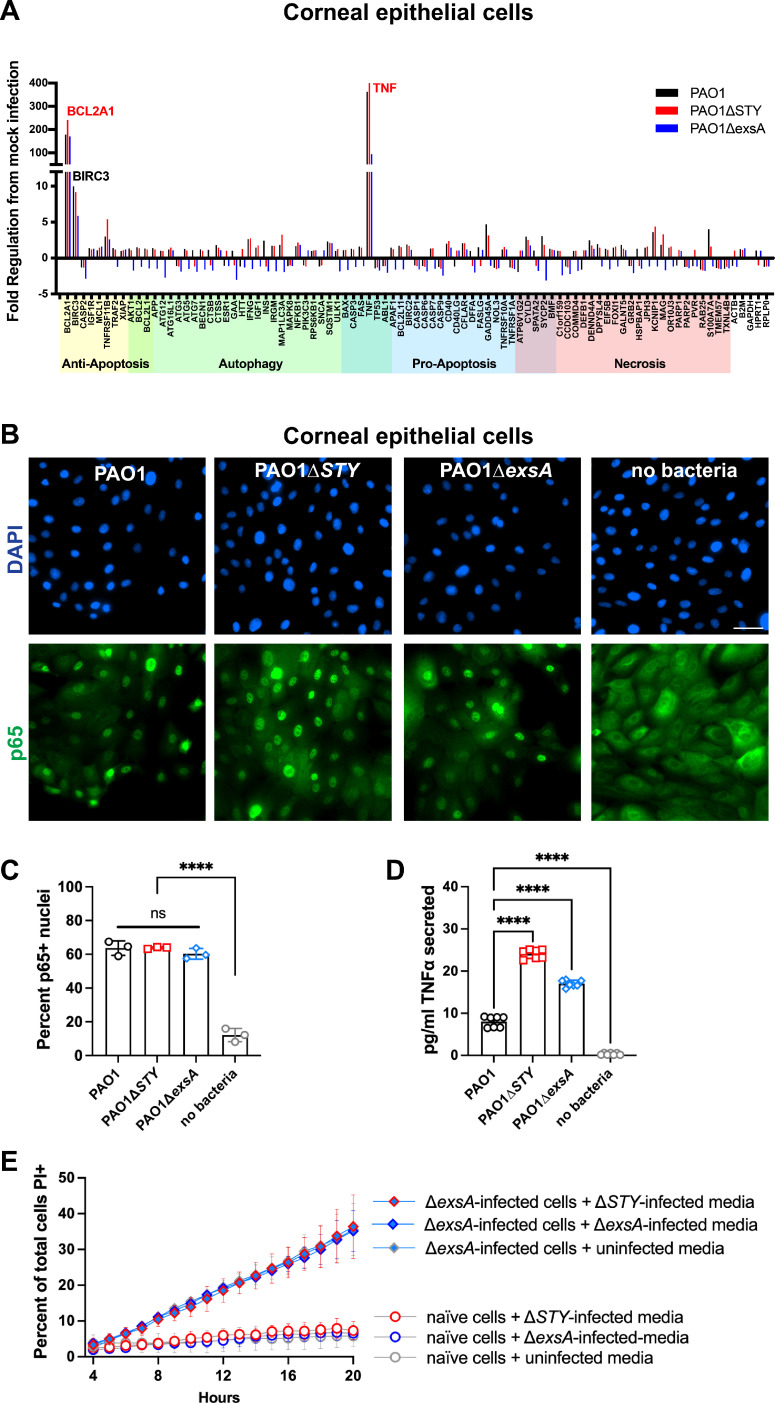
Corneal epithelial cell responses to *P. aeruginosa* mutants. (**A**) Corneal epithelial cells (hTCEpi) were infected with wild-type PAO1, PAO1∆*exoSTY*, or PAO1∆*exsA* for 3 h at an MOI equal to 10. Non-associated bacteria were removed, and media with amikacin was added for one additional hour. At 4 h, RNA was purified from infected cells, and RT-PCR analysis performed using a commercial array for genes associated with specific cell death pathways. (**B**) Corneal epithelial cells were infected as described in panel A. Cells were fixed at 4 h post-infection and p65 localization was determined using immunofluorescent staining. Nuclei were labeled with 4′,6-diamidino-2-phenylindole (DAPI). Scale bar = 50 µm. (**C**) Quantification of p65-positive nuclei. (**D**) Corneal epithelial cells were infected as described in panel A. Supernatant of infected cells was collected at 4 h post-infection and TNFα measured by enzyme-linked immunosorbent assay. (**F**) Supernatants of corneal epithelial cells infected with PAO1∆*exoSTY*, PAO1∆*exsA*, or uninfected were collected at 4 h post-infection and sterilized with 0.22 µm filter, and used as the replacement media on uninfected cells or PAO1∆*exsA*-infected cells at 3 h post-infection, combined with amikacin and propidium iodide. Hoechst was used to label all nuclei and propidium iodide used to label dead cells. The cell death rates were measured by time-lapse imaging, error bars display SD from three replicates.

To explore molecular mechanisms involved in *P. aeruginosa*-driven cell death from each mutant, a real-time PCR array with genes involved in regulated cell death pathways was used to study the candidate host responses. Results showed that tumor necrosis factor alpha (TNFα) and BCL2A1 were upregulated in excess of 55-fold by wild-type and by each mutant (Fig. 3A). Since TNFα and BCL2A1 can be regulated by nuclear factor (NF)-κB, the activation of NF-κB was measured by observing translocation of p65 to the nucleus (Fig. 3B). Infection with each tested strain caused p65 translocation, correlating with positive target gene transcription. Quantification of p65-positive nuclei was accomplished with an ImageJ macro (Fig. 3C). Since TNFα is associated with both apoptotic and necrotic cell death, TNFα secretion from infected cells was measured by enzyme-linked immunosorbent assay. The results showed that while wild-type PAO1 stimulated TNFα secretion from corneal epithelial cells, higher levels were detected after infection with the ∆*exoSTY* mutant, and the ∆*exsA* mutant causing an intermediate response (Fig. 3D). This is suggestive of a possible role of exotoxins in reducing cytokine expression or secretion post-transcriptionally.

Since TNFα secretion levels are lower in both wild-type PAO1 and ∆*exsA* infections relative to ∆*exoSTY* mutants, we next asked whether there was a causative relationship with cytokine secretion that might amplify cell death rates in response to ∆*exoSTY* mutants, or explain why some bystander cells are also killed. Thus, media from naïve cells or ∆*exsA* mutant-infected cells was replaced with filter-sterilized media from ∆*exoSTY* mutant-infected cells (high TNFα). The outcome showed that this did not enhance host cell death rates (Fig. 3E), suggesting cell death is not driven by a secreted factor such as TNFα, and requires the bacteria to be present.

### Caspase-4 is involved in cell death response to intracellular *P. aeruginosa*

The morphology of invaded cell death included membrane integrity loss, intact nucleus, and transient blebbing, all suggestive of pyroptosis (Fig. 2; Movie S1) (22). Since caspase-4 can restrict numerous other gram-negative bacteria (61, 64), and the pathogen *Shigella* inhibits the caspase-4 inflammasome by multiple mechanisms to survive (65 – 69), and corneal epithelial cells were shown to express caspase-4 (53), we tested the hypothesis that caspase-4 was involved in driving corneal epithelial cell death in response to intracellular *P. aeruginosa*. We generated *CASP4* knockout corneal epithelial cells, and loss of caspase-4 protein was confirmed by Western blot (Fig. S2). Control cell lines with nontargeting guide RNA were also generated using an identical selection strategy and grown up as monoclonal lines; each exhibited death rates consistent with wild-type, nontransduced hTCEpi cells (Fig. S3).

**Fig 4:**
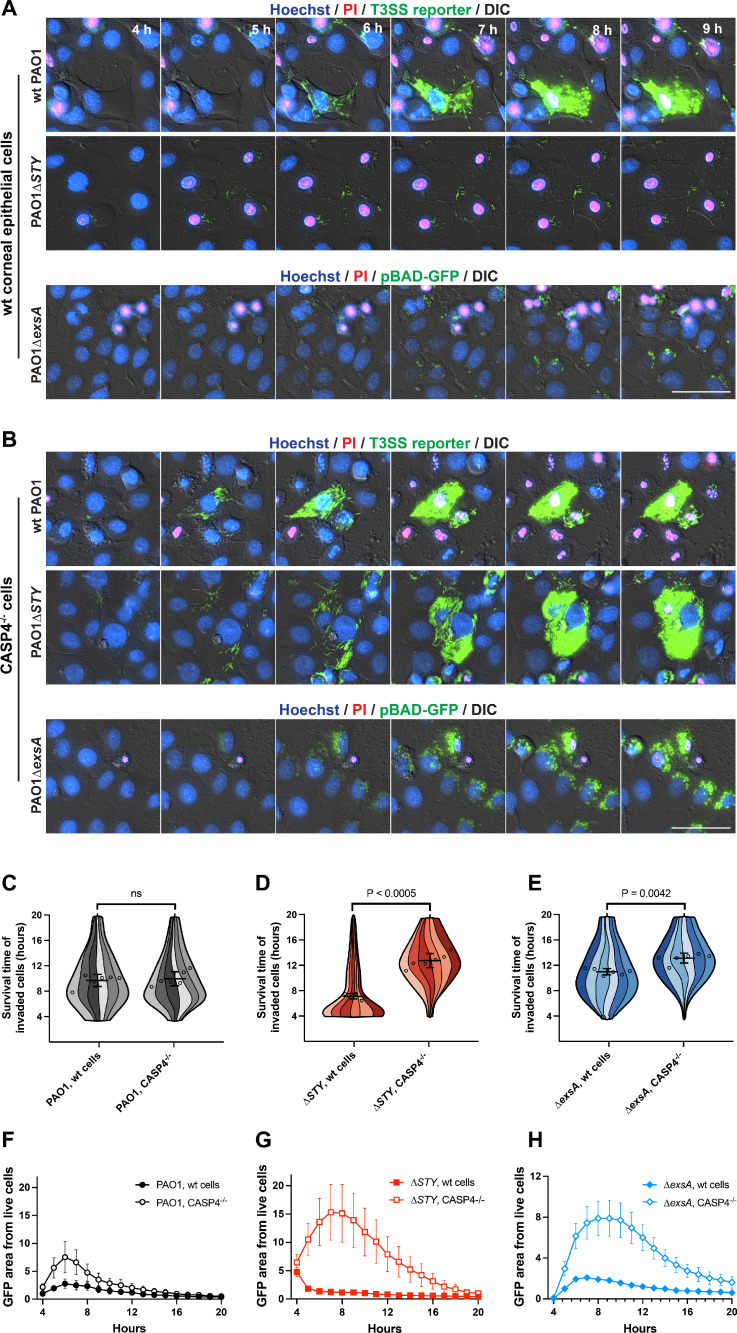
Caspase-4 limits intracellular replication and persistence of T3SS mutant bacteria. (**A**) Corneal epithelial cells (hTCEpi) or (**B**) cells knocked out for caspase-4 were infected with wild-type PAO1, PAO1∆*exoSTY* (each visualized by T3SS-GFP reporter) or PAO1∆*exsA* (pBAD-GFP induced) at an MOI of 10. Nuclei were labeled with Hoechst and propidium iodide. Extracellular bacteria were eliminated with amikacin at 3 h post-infection, and imaged hourly from 4 to 20 h post-infection. Images from 4 to 9 h post-infection are shown. Scale bar = 50 µm. Full-field time-lapses are available as Movie S2. (**C-E**) Cells from indicated infections were analyzed with a computational approach to segregate only invaded cells and measure survival times in hours. Six replicates were combined into a single super violin plot. Mean survival times with SD error bars are displayed, and significance determined by Student’s *t*-test. More than 900 cells were analyzed in each condition across six replicates. (**F-H**) GFP area was summed within the boundaries of live cells using the same data set as panels C-E, and normalized to PAO1 (wild-type) at 4 h post-infection.

Invasion by wild-type PAO1 yielded a similar cell death timing in both *CASP4* knockout cells as wild-type corneal epithelial cells (Fig. 4A through C). In contrast, ∆*exoSTY* mutant-invaded cells experienced significantly increased survival times (Fig. 4A, B and D), which allowed substantial accumulation of intracellular bacteria. A smaller but significant increase in invaded cell survival times was also observed with PAO1∆*exsA* mutant-occupied *CASP4* knockout cells (Fig. 4E). Bacterial replication was measured using summed GFP area contained in live cell masked region generated by the FIJI macro (19). Results indicate increased bacterial replication in *CASP4* knockout cells, with the most substantial increase observed in cells invaded by ∆*exoSTY* mutants (Fig. 4F through H). These data implicated the noncanonical inflammasome as a dominant response to cytoplasmic invasion of T3SS-positive *P. aeruginosa* independently of the T3SS effectors and also show involvement in responding to vacuole-occupying bacteria. Unchanged cell death timing for wild-type PAO1 in *CASP4* knockout cells aligns with our previously published data showing that the T3SS effector exotoxins, specifically the ADP ribosyltransferase activity of ExoS (19), counter cell death. Of note, while *CASP4* knockout reduced corneal epithelial cell death associated with intracellular ∆*exsA* mutants, many of the cells still died by the end of the 20-h imaging timeframe (Fig. 1 and 4E). This implies additional mechanisms for recognizing vacuolar/T3SS-negative *P. aeruginosa* in addition to caspase-4, which are also absent from HeLa cells.

Prior studies implicate Exotoxin S in delaying host cell lysis in response to invasion (19); thus, we examined the ∆*exoS* mutant in both wild-type corneal epithelial cells and *CASP4* knockout cells to evaluate whether there is a role for ExoT and ExoY in suppressing caspase-4-mediated lysis or supporting cytoplasmic bacterial growth (Fig. S4). Compared to the aforementioned study which tested strains encoding a single effector (e.g., ∆*exoST* or ∆*exoSY*) (19), the ∆*exoS* mutant showed a similar phenotype to ∆*exoSY* mutant, which expresses only ExoT: bacteria were unable to replicate substantially within in wild-type corneal epithelial cells (Fig. S4). Bacterial mutant replication was rescued in *CASP4* knockout cells. However, the timing of ∆*exoS*-invaded wild-type cell survival was irregular across the timeframe observed (Fig. S4C), which may relate to ExoT’s ability to block invasion (70, 71), or to interfere with other inflammasomes (41). However, ExoT and ExoY were unable to contribute to intracellular replication in cells with caspase-4.

### HeLa cell host responses are restricted to toxin-null cytoplasmic mutants

**Fig 5:**
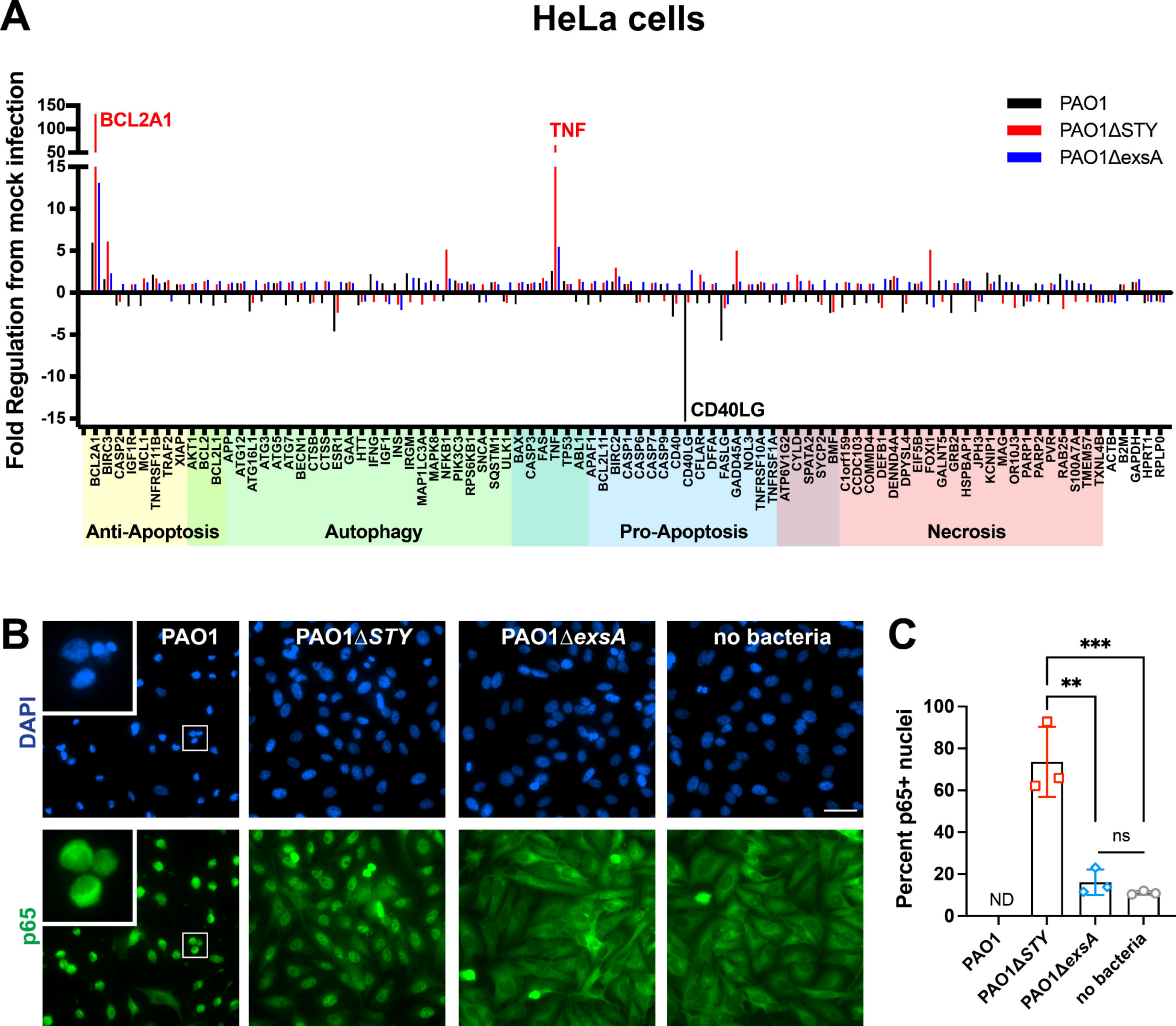
HeLa cell responses to *P. aeruginosa* mutants. (**A**) HeLa cells were infected with wild-type PAO1, PAO1∆*exoSTY*, or PAO1∆*exsA* for 3 h at an MOI equal to 10. Non-associated bacteria were removed, and media with amikacin was added for one additional hour. At 4 h, RNA was purified from infected cells, and RT-PCR analysis performed using a commercial array for genes associated with specific cell death pathways. (**B**) Corneal epithelial cells were infected as described in panel A. Cells were fixed at 4 h post-infection and p65 localization was determined using immunofluorescent staining. Nuclei were labeled with 4′,6-diamidino-2-phenylindole (DAPI). Scale bar = 50 µm. (**C**) Quantification of p65-positive nuclei. PAO1-infected HeLa cells were not able to be accurately quantified (indicated using “ND”) by automated analysis due to the small and irregular cytoplasmic area.

As a comparison to corneal epithelial cells, transcriptional responses and NF-κB activation status were measured in HeLa cells. In the transcriptional array, only the PAO1*∆exoSTY* mutant caused upregulation of TNFα and BCL2A1 (Fig. 5A). HeLa cells may lack an intrinsic response to extracellular or vacuolar ∆*exsA* mutants, which is present in corneal epithelial cells (Fig. 3A). The HeLa-specific response may be activated by either cytoplasmic location of bacterial bodies or the T3SS apparatus. Interestingly, the transcriptional response was absent in response to wild-type PAO1, suggesting that there is an exotoxin-mediated transcriptional suppression occurring only in HeLa cells. In addition, CD40LG was downregulated by wild-type bacteria in HeLa cells. Correlating with these results, only infection with ∆*exoSTY* mutants led to apparent p65 translocation in HeLa cells (Fig. 5B and C). Translocation of p65 in wild-type PAO1 infection was difficult to measure due to cell rounding and low cytoplasmic area; however, p65 appeared excluded from the nuclear region of HeLa cells (Fig. 5B, inset).

### IFN-γ stimulation enables HeLa cells to respond to intracellular *P. aeruginosa*

**Fig 6:**
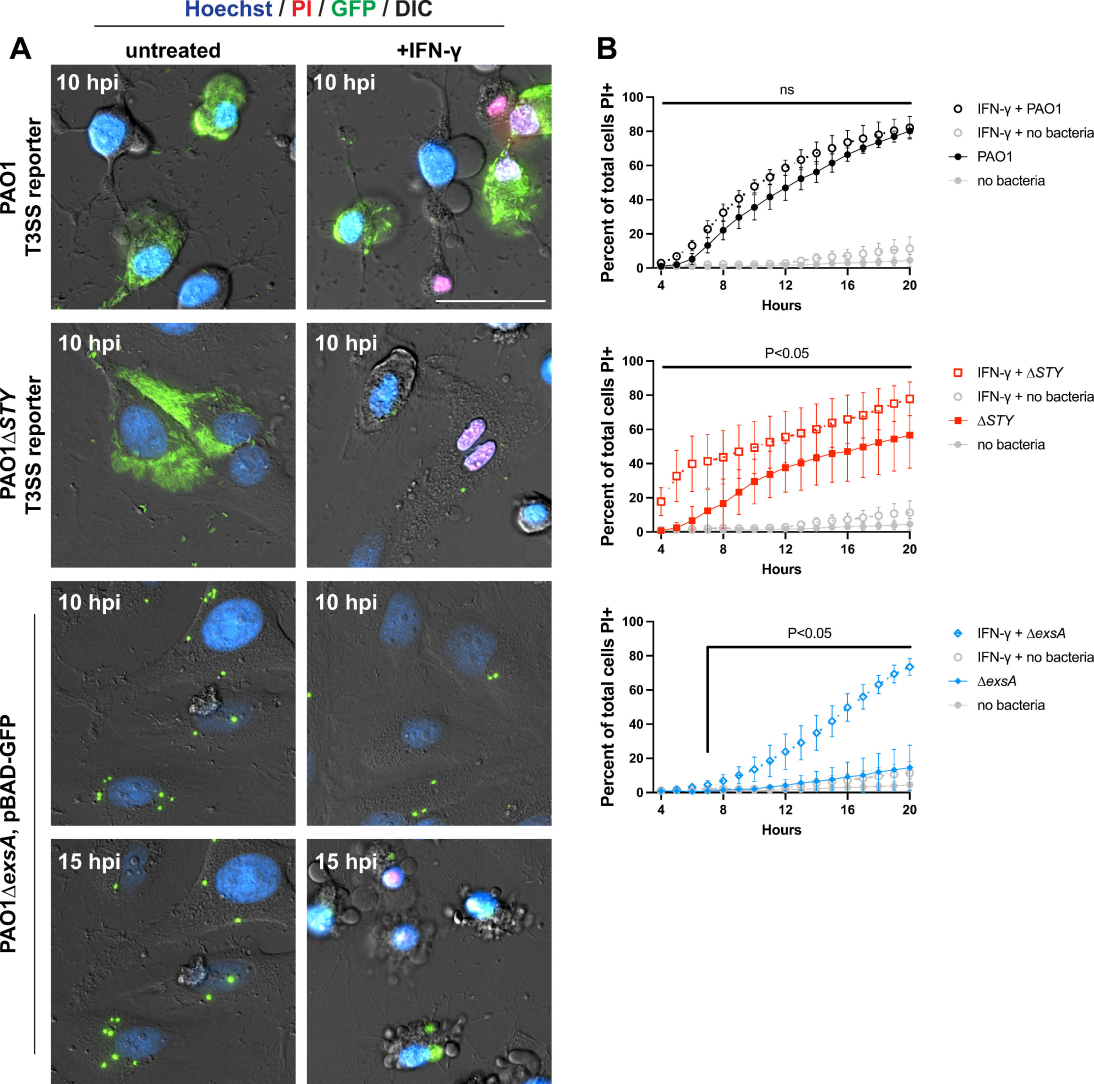
IFN-γ-stimulated HeLa cells are not permissive for PAO1∆*exoSTY* intracellular replication nor PAO1∆*exsA* intracellular persistence. (**A**) HeLa cells were treated with 50 ng/mL IFN-γ for 16 h prior to infection with wild-type PAO1, PAO1∆*exoSTY* (each using the T3SS-GFP reporter) or PAO1∆*exsA* (pBAD-GFP induced) at an MOI of 10. Nuclei were labeled with Hoechst and propidium iodide. Extracellular bacteria were eliminated with amikacin at 3 h post-infection, and imaged hourly from 4 to 20 h post-infection. Select timeframes are shown at either 10 or 15 h post-infection. Scale bar = 50 µm. Full-field time-lapses are available as Movie S3. (**B**) The rate of cell death was determined using host cell nuclear stains. All panels are from the same experiment with only indicated bacterial strain shown. Error bars show SD from three replicates. Multiple column *t*-tests were performed comparing IFN-γ-treated cells to untreated cells.

HeLa cells support cytoplasmic hyper-replication of *P. aeruginosa* exotoxin null mutants before significant impact on host cell viability occurs (3, 19). The bacterium *Salmonella* also accomplishes this within HeLa cells (72). Santos and colleagues demonstrated that IFN-γ stimulation causes HeLa cells to lyse in response to *Salmonella* entry into the cytosol, which was associated with upregulation of GBP proteins and stimulation of the caspase-4 inflammasome (61). We examined the impact of IFN-γ stimulation on HeLa cells containing intracellular *P. aeruginosa* (Fig. 6). The kinetics of wild-type PAO1-induced host cell death remained unchanged. However, the rate of cell death increased for both PAO1∆*exoSTY* and PAO1∆*exsA* infections (Fig. 1 and 6) (59). Aligning with the quick timing of cell lysis (Fig. 6B), PAO1∆*exoSTY* mutants were no longer able to replicate effectively in the cytoplasm of IFN-γ-treated HeLa cells (Movie S3). IFN-γ-treated HeLa cells infected by ∆*exsA* mutants gained a death response (Fig. 6B), reminiscent of corneal epithelial cells. Thus, IFN-γ-treated HeLa cells resembled corneal epithelial cells in their response to intracellular *P. aeruginosa*.

## DISCUSSION

Invasive (ExoS-expressing) strains of *P. aeruginosa* can thrive inside a wide range of host cells (2, 3, 5). After invading an epithelial cell, ExoS plays multiple roles in supporting intracellular survival and replication, including inhibition of host cell lysis (19). This study aimed to understand the underlying host response inhibited by ExoS. Knowing that *P. aeruginosa* diversifies intracellularly into vacuolar and cytoplasmic populations, bacteria in both locations trigger cell death, their locations are determined by T3SS expression state, and that host cell lysis can be suppressed by ExoS, we used T3SS mutants to interrogate mechanisms. Results showed that cell death in response to cytoplasmic *P. aeruginosa* lacking T3SS effectors involved caspase-4 and its deletion inhibits rapid pyroptotic lysis. Deletion of caspase-4 also partially alleviated cell death caused by bacteria lacking the T3SS that were restricted to vacuoles (∆*exsA* mutants). We further found that while HeLa cells lack this response to intracellular *P. aeruginosa*, it could be induced by IFN-γ, a stimulator of numerous genes including factors that assist in activation of the caspase-4 inflammasome (61). This result suggests that a critical difference between corneal epithelial cells and HeLa cells may be among interferon-stimulated genes (such as GBP1-4). For example, corneal epithelial cells might promptly upregulate inflammatory genes in response to *P. aeruginosa* or express these genes constitutively (Fig. 3). Another possibility is that corneal epithelial cells are sensitive to a different virulence factor produced by ∆*exsA* mutants, for example, proteases or phospholipases, and interferon stimulation confers a similar sensitivity to the HeLa cells. Since ExoS inhibits cell death in response to cytoplasmic bacteria (19), these findings implicate ExoS-mediated interference with the noncanonical inflammasome pathway. ExoS is capable of modulating cell survival to a similar timing as *ΔexsA* mutant-invaded cells, suggesting that it limits host response to T3SS-positive cytoplasmic bacteria, that is, it cannot compensate for cell death in response to T3SS-negative bacteria, which do not access the cytoplasm.

Since *P. aeruginosa* is often assumed an extracellular pathogen, previous studies of host epithelial cell death have been from the perspective of extracellular bacteria. In that regard, ExoS alone has cytotoxic activities. HeLa cells can undergo apoptotic cell death based on both caspase-3 (59) and caspase-8 activities (73), and the activation of apoptosis is through JNK1 and cytochrome c release (74). This was confirmed using a mammalian expression system for introducing ExoS in the absence of bacteria, which showed that it was sufficient to induce HeLa cell apoptosis (75). Placing our findings in the context of these earlier HeLa cell studies, we note differences in our experimental systems: while they explored mechanisms for cell death caused by ExoS, we studied cell death triggered by live intracellular bacteria lacking ExoS. If bacteria became internalized during these experiments, HeLa cells would not rapidly lyse since IFN-γ is not present. Our experiments also provide an analysis of only invaded cells excluding extracellular bacteria to focus on impact of intracellular bacteria, and used corneal cells that naturally respond to bacterial PAMPs. We then showed that IFN-γ-stimulated HeLa cells respond in the same manner: ExoS inhibits rather than drives a host cell death response. In sum, while introducing ExoS in isolation is a useful surrogate for *in vitro* experimentation to address specific questions, other factors modify the impact of ExoS in other situations. Despite differences in experimental models, it remains important to reconcile outcomes. The innate ability of ExoS to both drive and inhibit cell death, involving different types of regulated cell death, and correlating with the presence or absence of internalized *P. aeruginosa,* is interesting and worth exploring. Indeed, it could aid in determining mechanisms by which ExoS blocks pyroptosis, given crosstalk between pyroptosis and apoptosis (76).

The specific mechanism by which the ADPr activity of ExoS interferes with cell death (19), which we show here is caspase-4-mediated pyroptosis, remains to be identified. Recently, *Shigella flexneri* was shown to block the noncanonical inflammasome by multiple mechanisms: caspase-4 inactivation from ADP riboxanation by the effector OspC3 (65, 66), and ubiquitylating gasdermin D (77) and GBP1 (68) to target each for degradation. ExoS is unique among bacterial ADP ribosyltransferases for having numerous host substrates, though currently none are known to interact with the caspase-4/11 pathway (78). Since there are many possibilities to investigate, comprehensive identification of uncharacterized ExoS substrates and their potential involvement will require a separate study. Considering that *CASP4* knockout does not fully prevent cell death triggered by vacuole-bound T3SS mutants (Fig. 5), it would also be worth exploring which additional cell death pathways are involved.

In summary, the results of this study demonstrate a role for caspase-4 in limiting intracellular colonization of *P. aeruginosa* in ocular surface cells, a phenomenon triggered by the presence of intracellular bacteria but inhibited by the T3SS effector ExoS. Importantly, the data support the notion that *P. aeruginosa* can function as both an intracellular and an extracellular pathogen, suggestive of a T3SS effector function devoted to inhibition of an *intracellular* pattern recognition receptor, consistent with its unusual capacity to adapt to its environment, survive hardship, and exist ubiquitously. Ultimately, the development of effective strategies to prevent or treat the devastating infections caused by *P. aeruginosa* will require a thorough appreciation of these complexities.

## MATERIALS AND METHODS

### Bacterial strains and cell lines

*P. aeruginosa* strain PAO1 and isogenic mutants of *exsA*, ExoS/ExoT/ExoY, or ExoS were used for all infection experiments (79). Plasmids used for visualization were pJNE05 (80) and pBAD-GFP (58). Plasmid selection was achieved with 100 µg/mL gentamicin. Corneal epithelial cells (hTCEpi) (54) were maintained in the KGM-2 media (Lonza) lacking gentamicin. HeLa cells were maintained in the phenol red-free Dulbecco’s Modified Eagle Medium (DMEM; Gibco) with 10% fetal bovine serum (FBS).

### CRISPR-Cas9 knockout cell line

Lentiviral particles for transduction were generated from 293T cells transfected with psPAX2 (Didier Trono, Addgene plasmid # 12260) and pMD.2 (Didier Trono, Addgene plasmid # 12259), combined with either a Cas9 expression vector plenti-Cas9-blast (Feng Zhang, Addgene plasmid # 52962) (81) or a guide RNA vector plenti-LIC-Puro which was adapted for ligation-independent cloning (gifted by Moritz Gaidt) (82). Guide RNA sequences targeting caspase-4 (5′-CCACAGAAAAAAGCCACTTA-3′) or a nontargeting control sequence (5′-GACGGAGGCTAAGCGTCGCA-3′) were cloned into plenti-LIC-Puro using ligation-independent cloning. After 6 h, media on 293T cells was replaced with KGM-2. Media was collected at 48 h and passed through a 0.45-µm filter and added directly to hTCEpi cells, which were then centrifuged at 1200 × *g* for 90 min at 37°C. Antibiotic selection with blasticidin (50 µg/mL) or puromycin (10 µg/mL) was performed after 2 d. For the generation of monoclonal lines, single cells were seeded in 96-well plates. Isolated colonies were identified and grown up over approximately 12 d before transferring to 6-well plates to avoid differentiation induced by crowding. *CASP4* knockout candidates were screened by Western blot: lysates from a 6-well plate were collected in a 150 µL RIPA buffer, frozen and thawed, and then separated by centrifugation for 20 min at 4°C. Samples were run on 10% stain-free gels (Bio-Rad) and the total protein visualized as a loading control prior to transferring to a 0.2-µm polyvinylidene difluoride membrane. Membrane was blocked with Bio-Rad EveryBlot block and probed with anti-caspase-4 antibody (Santa Cruz, sc-56056, 1:1000). Blot was washed using Tris-buffered saline with 0.1% Tween-20, and the antibody was detected using the goat anti-rabbit HRP (Bio-Rad, 1706515, 1:5000).

### Infection experiments

One day preceding experiments, hTCEpi cells were plated on No. 1.5 glass-bottom 24-well plates (MatTek) in KGM-2 at 75% confluence with 1.15 mM calcium to induce differentiation (54). HeLa cells were maintained in DMEM with 10% FBS and plated at 50% confluence on optical plastic 8-well chambered coverslips (ibidi). Where indicated, 50 ng/mL IFN-γ (Peprotech) was added at 16 h prior to infections of HeLa cells and maintained throughout the infection and imaging.

Bacterial suspensions were made in phosphate-buffered saline (PBS) from 16-h lawns grown on tryptic soy agar (TSA) media at 37°C. The absorbance at 540 nm was measured, and MOI of 10 was calculated using OD540 of 1 equal to 4 × 10^8^ CFU per mL. Bacteria were directly to cell culture media and allowed to infect for 3 h. For live imaging, Hoechst (0.8 µg/mL) was added to the cells prior to infections and allowed to label the cells during the 3-h infection period. Media was replaced with media containing the antibiotic amikacin (200 µg/mL) and propidium iodide (0.8 µg/mL). Where needed, a 1:10 dilution of 10% arabinose in media was added at 3.75 h post-infection. Typical levels of bacterial internalization ranged from ~20–40% of total cell numbers and was similar for wild-type PAO1 and its T3SS mutants (data not shown).

### RT-PCR arrays

Cells in 10 cm dishes were infected with PAO1, PAO1∆*exoSTY*, or PAO1∆*exsA* for 3 h at an MOI of 10, and media was replaced for 1 h with 200 µg/mL amikacin. At 4 h, cells were washed twice in PBS and collected in 1 mL TriReagent (Sigma). RNA was purified using Zymo Direct-zol RNA MiniPrep. cDNA synthesis was performed with the RT2 first strand kit (Qiagen) using 0.5 µg RNA. RT-PCR was performed on a Roche LightCycler 96 using the RT² Profiler PCR Array Human Cell Death PathwayFinder (PAHS-212Z, Qiagen), and analyzed in Qiagen’s Data Center Analysis Portal (https://geneglobe.qiagen.com/us/analyze). Arrays were performed once for each condition.

### Fixed immunostaining

Cells were seeded on No. 1.5 glass coverslips and infected as described above. At 4 h post-infection, cells were washed twice in PBS and fixed in 4% paraformaldehyde in PBS for 10 min. Cells were washed twice in PBS, and neutralized with 150 mM glycine in PBS for 10 min. Cells were washed twice in PBS, and permeabilized/blocked (5% FBS, 2.5% cold fish skin gelatin, 0.1% Triton X-100, and 0.05% Tween-20 in PBS) for 1 h. Antibody in solution (2.5% FBS, 1.25% cold fish skin gelatin, 0.1% Triton X-100, and 0.05% Tween-20 in PBS) was incubated overnight at 4°C. Cells were washed four times (5 min), and secondary antibodies were added to antibody solution for 1 h. Cells were washed once, labeled with 4′,6-diamidino-2-phenylindole for 5 min, and washed twice (5 min). Coverslips were mounted using the ProLong Diamond.

### Microscopy

Images for Fig. 1, Fig. 4A and B were captured on a Nikon Ti-E inverted microscope equipped with a Lumencor Spectra X LED Light Engine illumination source. All other images were captured on a Ti2-E inverted microscope with X-Cite XYLIS XT720S Broad Spectrum LED Illumination System. Both systems used Nikon perfect focus, an Okolab stage-top incubation chamber, a DS-Qi2 CMOS camera, and a CFI Plan Apochromat Lambda D 40X air NA 0.95 objective. Time-lapse fields were selected between hours 3 and 4 without observing fluorescence channels to limit bias in field selection. Eight fields per condition were imaged hourly from 4 to 20 h post-infection.

### Image analysis

Time-lapse images were computationally analyzed using two custom macros written for the FIJI package of Image J, as previously described (19). The code for measuring the rates of cell death for the whole population and set of macros for tracking the invasion state of cells is available in a GitHub repository: https://github.com/Llamero/Nuclei_analysis-macro. Subsequent Python scripts for exporting and processing the analysis of TIF files are available in the following GitHub repository: https://github.com/abbykroken/cell_survival_with_bacteria. An Image J macro for measuring a ratio of p65 stain intensity within the nucleus and the periplasmic region has been made available in the following GitHub repository: https://github.com/abbykroken/Nucleus-to-cell-ratio.

### *In vitro* T3SS effector secretion

Bacteria were grown for 5 h in 5 mL of TSB supplemented with 100 mM MSG and 1% glycerol at 37°C with shaking at 200 rpm. Ethylene glycol-bis(β-aminoethyl ether)-N,N,N′,N′-tetraacetic acid (EGTA, 2 mM) was added to induce T3 secretion. OD readings of 540 nm were taken prior to supernatant protein concentration and used to normalize volumes. Bacteria were centrifuged at 12,000× *g* to separate 1 mL of supernatant. Proteins were concentrated using trichloroacetic acid (TCA) precipitation: 250 µL of 100% cold TCA was added to 1 mL of supernatant for 30 min and centrifuged at 14,000× *g* for 5 min. The pellet was washed with 1 mL of cold acetone twice and suspended in 4× laemmli buffer normalized to starting OD (max volume 50 µL). Proteins were visualized using 10% stain-free gels (Bio-Rad).

### Statistics

Statistical analyses were performed and the data were presented using Graph Pad Prism 9. Super violin plots were prepared using scripts published by Kenny and Schoen (63) and output images aligned on axes generated in Graph Pad Prism 9. Data were shown as a mean ± standard deviation (SD) of 3–6 independent experiments unless otherwise indicated. Comparison of two groups was performed by Student’s *t*-test, three or more groups by a one-way ANOVA with Tukey’s post hoc analysis. Comparison between two groups for total cell death rates over time was performed by multiple column *t*-tests for each timepoint, and the two samples that were compared are specified in the figure legends. In each instance, *P < 0.05, **P < 0.01, ***P < 0.005, and ****P < 0.001.

## SUPPLEMENTAL MATERIAL

Supplemental Figures and Legends msphere.00351-23-s0001.pdf Fig. S1 to S3 and legends for the supplemental movies.

Movie S1 msphere.00351-23-s0002.mp4 Corneal epithelial cells invaded by *P. aeruginosa* and mutants. Corneal epithelial cells (hTCEpi) were infected with wild-type PAO1, PAO1∆*exoSTY* (each expressing GFP by T3SS reporter), or PAO1∆*exsA* (inducible GFP) for 3 hours at an MOI equal to 10. Then media was replaced with amikacin-containing media. Arabinose was added to PAO1∆*exsA* condition at 3.5 hours post infection. Imaging was conducted hourly from 4 to 20 hours.

Movie S2 msphere.00351-23-s0003.mp4
*CASP4* knockout corneal epithelial cells invaded by *P. aeruginosa* and mutants. Corneal epithelial cells (hTCEpi) or *CASP4* knockout cells were infected with wildtype PAO1, PAO1∆*exoSTY* (each expressing GFP by T3SS reporter), or PAO1∆*exsA* (inducible GFP) for 3 hours at an MOI equal to 10. Then media was replaced with amikacin-containing media. Arabinose was added to PAO1∆*exsA* condition at 3.5 hours post infection. Imaging was conducted hourly from 4 to 20 hours.

Movie S3 msphere.00351-23-s0004.mp4 IFN-γ-stimulated HeLa cells invaded by *P. aeruginosa* and mutants. HeLa cells were treated with 50 ng/ml IFN-γ for 16 hours prior to infection with wild-type PAO1, PAO1∆*exoSTY* (each using the T3SS-GFP reporter) or PAO1∆*exsA* (pBAD-GFP induced) at an MOI of 10. Nuclei were labeled with Hoechst and Propidium iodide. Extracellular bacteria were eliminated with amikacin at 3 hours post infection, and imaged hourly from 4 to 20 hours post infection. Arabinose was added to PAO1∆*exsA* condition at 3.5 hours post infection. Imaging was conducted hourly from 4 to 20 hours. IFN-γ was maintained during infection and imaging.

ASM does not own the copyrights to Supplemental Material that may be linked to, or accessed through, an article. The authors have granted ASM a non-exclusive, world-wide license to publish the Supplemental Material files. Please contact the corresponding author directly for reuse.
