# Supplementary material for: Intracellular replication of Pseudomonas aeruginosa in epithelial cells requires suppression of the caspase-4 inflammasome
Source: mSphere. 2023 Aug 17;8(5):e00351-23. doi: 10.1128/msphere.00351-23 (PMC10597407; doi:10.1128/msphere.00351-23)
Supplement: Supplemental Figures and Legends — Fig. S1 to S3 and legends for the supplemental movies. [file msphere.00351-23-s0001.pdf]

Supplemental Figure 1

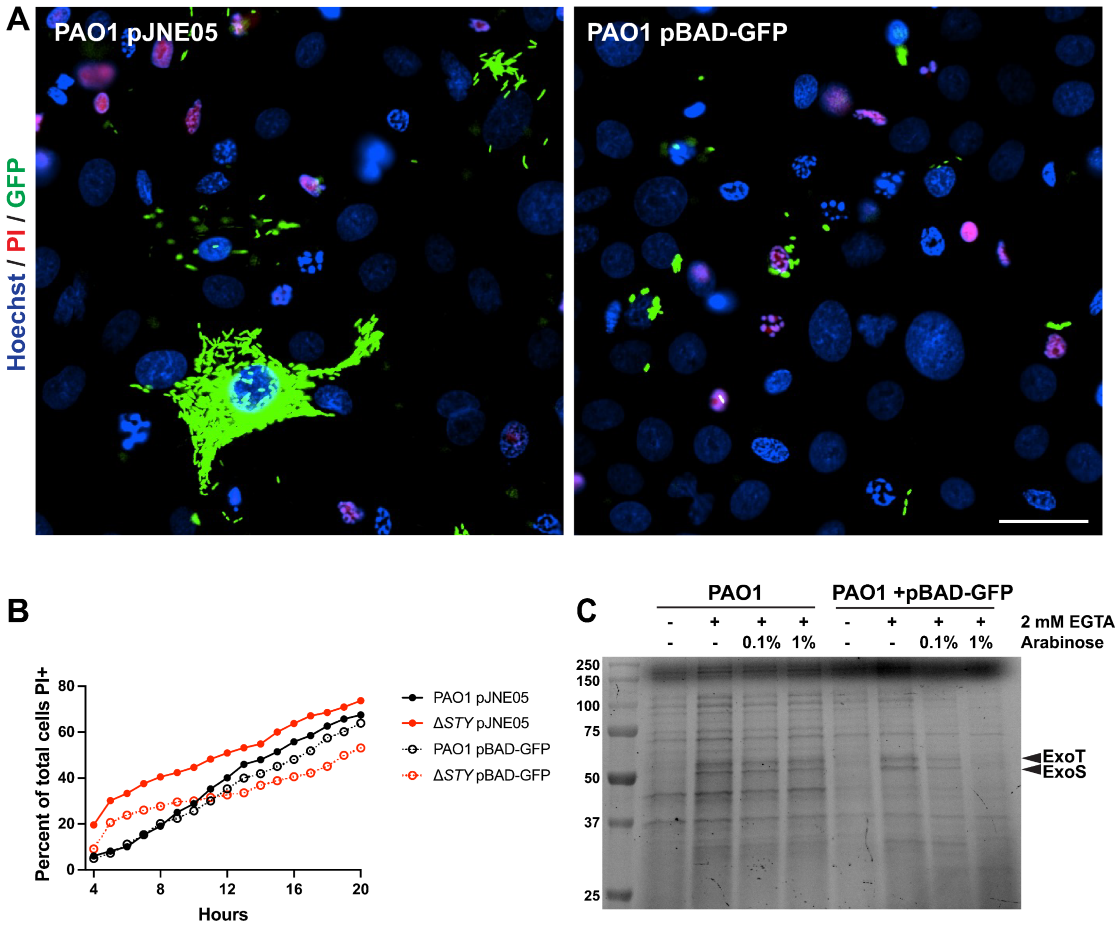

Supplemental Figure 1. GFP expression from plasmid pBAD-GFP suppresses the T3SS. (A) wild-type PAO1 was transformed with the T3SS reporter plasmid pJNE05, or the GFP inducible plasmid pBAD-GFP and imaged hourly in time-lapse. Example fields are shown in which bacteria expressing GFP under the T3SS reporter exhibit intracellular spread and replication, whereas those with inducible GFP show only limited intracellular replication. Scale bar = 50 µm. **(B)** The cell death rates of wild-type PAO1 or PAO1Δ*exoSTY* expressing GFP by either plasmid were measured, showing a trend of reduced cell killing rates for PAO1Δ*exoSTY* when GFP expression is induced. **(C)** wild-type PAO1 with indicated plasmid was grown for 5 hours in LB media with EGTA to induce the T3SS, and arabinose to induce GFP. Supernatant proteins were concentrated by TCA precipitation, and the bands constituting ExoS and ExoT are indicated.

Supplemental Figure 2.

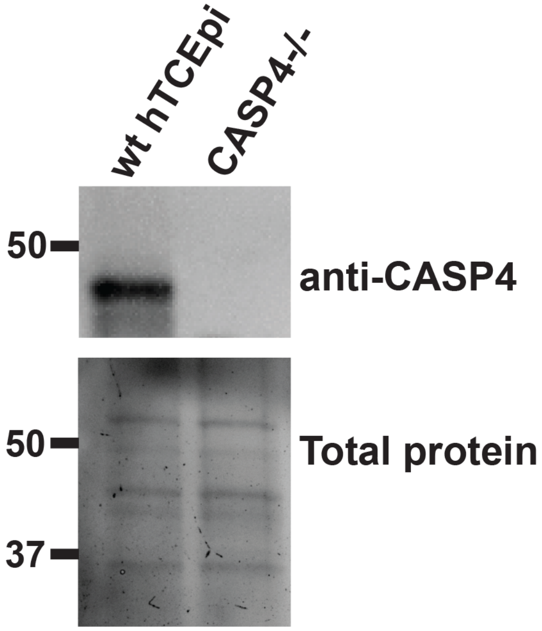

Supplemental Figure 2. Confirmation of caspase-4 deletion. Lysates from wild-type hTCEpi cells or caspase-4 candidate knockout cells were collected in RIPA buffer and clarified. Total protein was visualized using Bio-Rad stain free gels prior to transfer. Blot was probed with anti-caspase-4 antibody.

Supplemental Figure 3

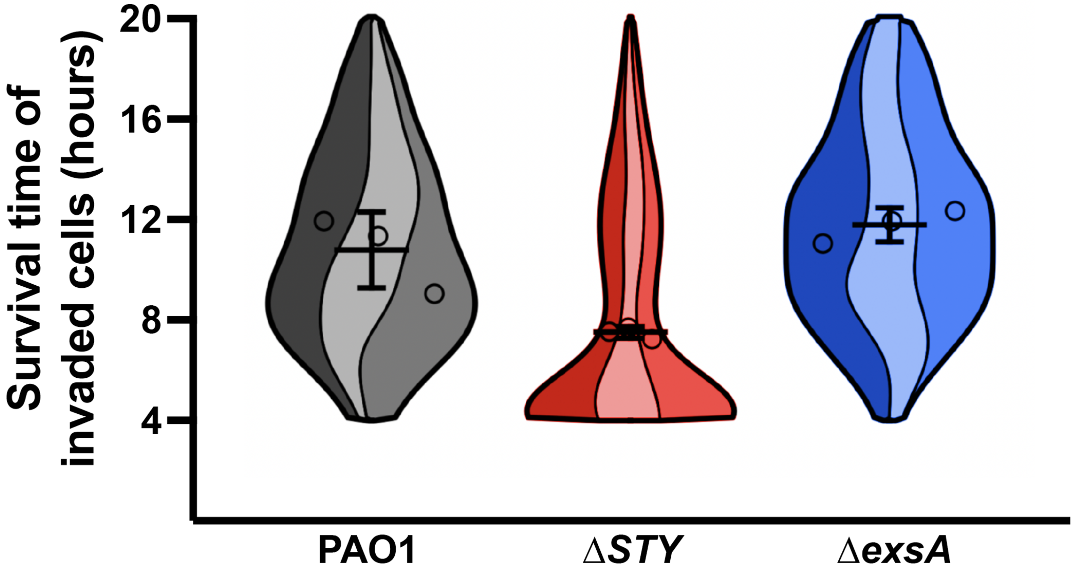

Supplemental Figure 3. Control monoclonal cells for CRISPR-Cas9 mutagenesis. A control cell line was generated using non-targeting guide RNA, and monoclonal line generated. Cells were infected with wild-type PAO1, PAO1Δ*exoSTY* (each using the T3SS-GFP reporter) or PAO1Δ*exsA* (pBAD-GFP induced) at an MOI of 10. Nuclei were labeled with Hoechst and Propidium iodide. Extracellular bacteria were eliminated with amikacin at 3 hours post infection, and imaged hourly from 4 to 20 hours post infection. Images were analyzed with a computational approach to segregate invaded cells and measure survival times in hours. Three replicates were combined into a single super violin plot. Greater than 450 cells were analyzed in each condition across all three replicates. Mean survival times with SD error bars are displayed.

Supplemental Figure 4

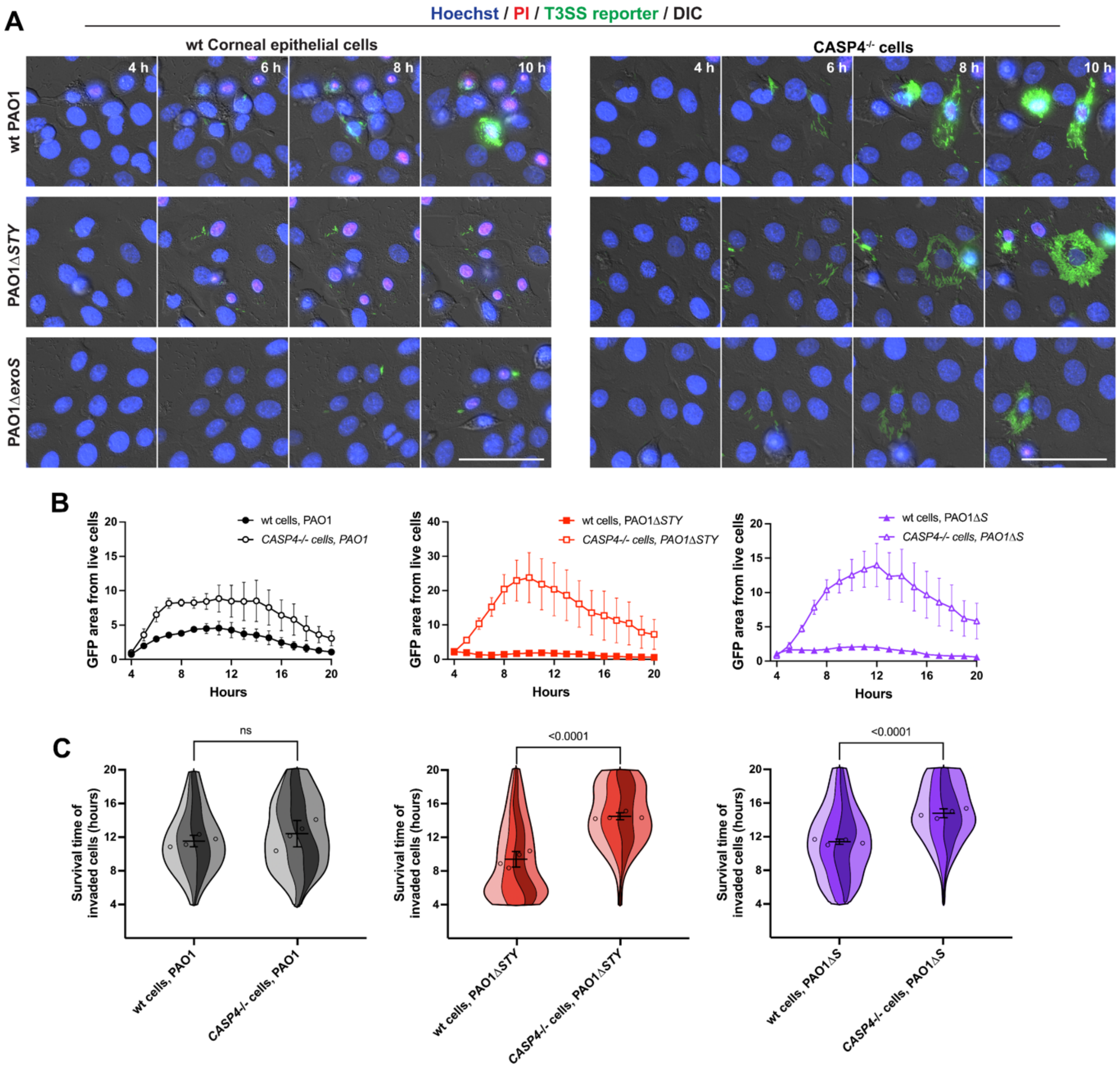

Supplemental Figure 4. Host cell survival time and intracellular replication of single exotoxin deletion of ExoS. (A) Corneal epithelial cells or cells lacking caspase-4 were infected with wild-type PAO1, PAO1Δ*exoSTY*, or PAO1Δ*exoS* (each visualized by T3SS-GFP reporter) at an MOI of 10. Nuclei were labeled with Hoechst and propidium iodide. Extracellular bacteria were eliminated with amikacin at 3 hours post infection, and imaged hourly from 4 to 20 hours post infection. Images from 4-10 hours are shown. Scale bar = 50 µm. **(B)** GFP area was summed within the boundaries of live cells and normalized to PAO1 (wild-type) at 4 hours post infection. **(C)** Cells from indicated infections were analyzed with a computational approach to segregate only invaded cells and measure survival times in hours. Greater than 600 cells were analyzed in each condition across all four replicates. Mean survival times with SD error bars are displayed, and significance determined by Student's t-test.

Supplemental Movie 1. Corneal epithelial cells invaded by *P. aeruginosa* and mutants. Corneal epithelial cells (hTCEpi) were infected with wild-type PAO1, PAO1Δ*exoSTY* (each expressing GFP by T3SS reporter), or PAO1Δ*exsA* (inducible GFP) for 3 hours at an MOI equal to 10. Then media was replaced with amikacin-containing media. Arabinose was added to PAO1Δ*exsA* condition at 3.5 hours post infection. Imaging was conducted hourly from 4 to 20 hours.

Supplemental Movie 2. *CASP4* knockout corneal epithelial cells invaded by *P. aeruginosa* and mutants. Corneal epithelial cells (hTCEpi) or *CASP4* knockout cells were infected with wild-type PAO1, PAO1Δ*exoSTY* (each expressing GFP by T3SS reporter), or PAO1Δ*exsA* (inducible GFP) for 3 hours at an MOI equal to 10. Then media was replaced with amikacin-containing media. Arabinose was added to PAO1Δ*exsA* condition at 3.5 hours post infection. Imaging was conducted hourly from 4 to 20 hours.

Supplemental Movie 3. IFN-γ-stimulated HeLa cells invaded by *P. aeruginosa* and mutants. HeLa cells were treated with 50 ng/ml IFN-γ for 16 hours prior to infection with wild-type PAO1, PAO1Δ*exoSTY* (each using the T3SS-GFP reporter) or PAO1Δ*exsA* (pBAD-GFP induced) at an MOI of 10. Nuclei were labeled with Hoechst and Propidium iodide. Extracellular bacteria were eliminated with amikacin at 3 hours post infection, and imaged hourly from 4 to 20 hours post infection. Arabinose was added to PAO1Δ*exsA* condition at 3.5 hours post infection. Imaging was conducted hourly from 4 to 20 hours. IFN-γ was maintained during infection and imaging.
